# Investigation of the 4D Multi-Material 316L/FeNi36 Obtained by Selective Laser Melting

**DOI:** 10.3390/mi15111288

**Published:** 2024-10-23

**Authors:** Arseniy Repnin, Evgenii Borisov, Aleksey Maksimov, Daria Rozhkova, Anatoly Popovich

**Affiliations:** Institute of Machinery, Materials, and Transport, Peter the Great St. Petersburg Polytechnic University (SPbPU), Polytechnicheskaya, 29, 195251 Saint Petersburg, Russia

**Keywords:** selective laser melting, 4D multi-material, bio-inspired structure, 316L, FeNi36

## Abstract

Multi-material can have functional properties, which are not typical for the materials of which they are composed (for instance, shape-changing effect). This can be used in robotics, micromachines, aerospace, and other fields. In this work, the 316L/FeNi36 multi-material produced by selective laser melting was investigated. The results show that the interfacial zone of the multi-material exhibits mixing regions of the two alloys but no defects. The microstructure is constituted by large grains with epitaxial growth, which propagate in a directional manner from the 316L alloy through the interfacial zone to the FeNi36 region. The multi-material sample displays three different zones of chemical composition: the FeNi36 composition zone; the interfacial zone; and the 316L zone. The size of the interfacial zone is approximately 50 µm. The multi-material sample exhibits the presence of three distinct phases: γ-Fe; γ-Fe64Ni36; and α-Fe. The hardness of the FeNi36 zone is approximately 163 HV, followed by an interfacial zone with a hardness of approximately 200 HV and then, the 316L zone with a hardness of approximately 214 HV. Functional tests demonstrate that the shape-changing effect is directly correlated with the variation in the FeNi36 thermal expansion coefficient with temperature. For achieving the most pronounced shape-changing effect, the temperature range of 25–215 °C is more suitable.

## 1. Introduction

Nature provides mankind with a vast array of inspiration in numerous areas of life through the utilization of well-coordinated, designed, and created processes, algorithms, materials, and structures [1]. Bio-inspired materials, as defined by the scientific community, are materials that allow for the mimicking of the natural structures inherent to living organisms [2]. These materials exhibit distinctive functional and strength properties, reliability, durability, and other advantageous characteristics. This provides the motivation for the development of innovative structural and functional materials for diverse practical applications. For example, an effective anti-corrosion coating inspired by the structure of gecko legs, which exhibit high adhesion, has been developed [3]. Another example is the scales of the fish Arapaima gigas, which serve as natural body armor with excellent mechanical properties and superior flexibility. Spider silk, nacre, and collagen fibers of the helicoidal structure found in shrimp claws all exhibit excellent impact strength [4]. Various plants (Bauhinia pods, flowers, etc.) and animals also exhibit shape-shifting properties that help them survive and reproduce [5].

Smart bio-inspired materials represent a class of materials that can respond to external stimuli and adapt to changing environmental conditions. Therewith, the structure of these materials is close to that observed in natural systems [6]. An illustrative example of a material that exhibits shape-changing properties is the nickel–titanium alloy (Nitinol, NiTi) [7]. In addition, there are polymers with a similar effect [8] and ceramic materials (piezoceramics) [9]. The distinctive characteristics of shape memory materials suggest a promising potential for their integration into a diverse array of novel applications, including mobile robots, micromachines, wearable devices, aerospace/automotive components, and biomedical devices [10]. However, to achieve the desired shape-changing effect, it is sometimes necessary to utilize not only smart materials but also to create a sophisticated bio-inspired structure. It is a challenge to reproduce the intricate structure of natural materials using conventional manufacturing techniques. Nevertheless, the development of new technologies, particularly additive manufacturing, is enabling the effective manufacturing of intricate structures at different scales [11].

Additive manufacturing (AM, 3D-printing) is a process of synthesizing (bonding, sintering, cladding, melting, etc.) materials to create products based on a digital model, usually layer by layer, as opposed to subtractive manufacturing methods [12]. This technology offers a number of advantages, including the freedom from technological constraints inherent in the design process, the acceleration of new product production, the reduction in costs associated with the manufacture of complex products on a small scale, the creation of personalized products, the minimization of the technological operations, the reduction in supply chains, and improving the environmental sustainability of production [13]. AM can be classified into seven groups: binder jetting; directed energy deposition; material extrusion; sheet lamination; material jetting; vat photopolymerization; and powder bed fusion [14]. The materials used in AM can be grouped into three main categories: polymers; metals; and ceramics. The two most common metal AM methods are directed energy deposition (DED) and powder bed fusion (PBF) [15]. Selective laser melting (SLM) is a type of PBF in which a laser beam is employed to melt metal powders layer by layer according to the 3D model.

Four-dimensional printing is an innovative manufacturing technology that enables the creation of smart materials and structures through the use of AM [16]. The current state of research in this field is promising, with a significant number of studies underway. In a recent study, researchers employed the SLM to produce NiTi samples of varying thicknesses (0.15–1.00 mm) at different scan speeds (500–1200 mm/s). They investigated the phase transformations and mechanical properties of these samples over a range of thicknesses [17]. In another paper, the effect of heat treatment on NiTi samples produced by the SLM was conducted. The microstructure and functional properties were investigated. One of the samples demonstrated superelasticity with a recoverable strain of 7% and a superelasticity temperature range of approximately 100 °C [18]. It is noteworthy that the principle of shape-changing can be achieved by utilizing bio-inspired structure, which involves a change in the chemical composition of the material. Natural examples of such structures include the pine cone [19] and plant petals [20].

There are already existing examples of materials that have been developed with bio-inspired structures and exhibit a change in chemical composition. These include functional-gradient materials and multi-materials, as well as coatings. The latter have a more limited application in the manufacture of products with complex geometries. The first two types have significant potential for investigation and use in the industry [21,22]. The prospects of multi-materials obtained by SLM are particularly promising [23]. There are multiple methods for conducting multi-material printing by SLM. The first method is relatively straightforward and involves depositing one material on top of another, resulting in a change in chemical composition in a single direction [22]. The second method is more complex and involves printing materials with a change in chemical composition in three directions.

The utilization of bio-inspired structures and multi-material AM enables the generation of new functional materials. The unique feature of these materials is that, separately, they do not have functional properties, but when they are combined in accordance with the nature-like structure, such a possibility arises. It is worth noting that there is a lack of research on multi-materials with shape-changing properties. The available studies are primarily focused on polymers, as they are more easily obtained from a technological standpoint [24]. Nevertheless, there are promising opportunities for the development of metallic multi-materials with functional properties, which could potentially be utilized in a range of sectors, including robotics, aviation, the automotive industry, etc. Currently, there is a growing interest in research on the multi-materials based on stainless steel, titanium, and nickel alloys obtained by the SLM [16]. Nevertheless, multi-materials in which functional properties can be realized remain the subject of limited research. Examples of such multi-materials are 316L/FeNi36, FeNi36/NiTi, NiTi/316L, etc. The realization of their functional properties can be achieved by creating a bio-inspired structure, for instance, by utilizing materials with different thermal expansion coefficients. The use of NiTi in multi-materials is associated with the formation of defects in the interfacial zone due to the presence of brittle intermetallic compounds and differences in thermophysical properties [25]. The 316L/FeNi36 multi-material, which has already been investigated using other AM methods [26], can be considered for research. Accordingly, the objective of this study was to examine the 316L/FeNi36 multi-material derived from a functional bio-inspired structure. The microstructure, chemical and phase compositions, and hardness of the multi-material samples were investigated. Furthermore, functional tests and computer modeling of the shape-change effects were conducted.

## 2. Materials and Methods

### 2.1. Starting Materials

**Figure 1:**
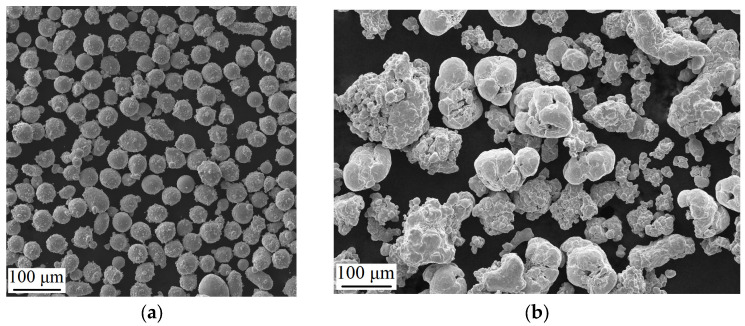

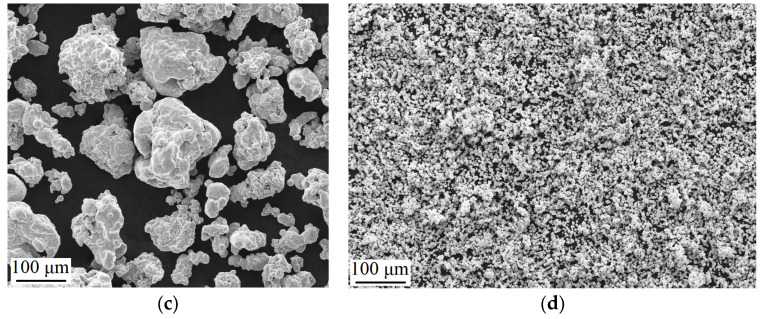
The morphology of the powders used for the fabrication of multi-material by the SLM: (**a**) 316L alloy powder; (**b**) mixed FeNi36 powder; (**c**) Fe powder; (**d**) Ni powder.

The functional properties of the 316L/FeNi36 multi-material will be achieved due to the presence of two materials with different coefficients of thermal expansion (CTE) in one sample. Two types of powder were used to fabricate the 316L/FeNi36 multi-material samples: spherical 316L powder (SferaM LLC, Chelyabinsk, Russia, Figure 1a) and non-spherical FeNi36 powder (Figure 1b). The FeNi36 powder was obtained by mixing pure iron (BVB-Alliance LCC, Russia, Figure 1c) with pure nickel (BVB-Alliance LCC, Moscow, Russia, Figure 1d) in the proportion 64 wt.% Fe and 36 wt.% Ni. The presence of the FeNi36 non-spherical powder mixture may result in a reduction in technological capability, although this should not significantly impact the printing quality. It is also important to note that the particle size distribution of the powders is different. The particle size of 316L steel is 10–63 µm, and iron powder is 45–100 µm. However, the size of nickel powder is less than 10 µm. This can be beneficial when mixing the powders, as it will facilitate a more uniform distribution of Ni in the powder mixture with Fe.

### 2.2. L-PBF Process Parameters and the 316L/FeNi36 Multi-Material Samples

The 316L/FeNi36 multi-material samples were produced by the 3DLam Mini SLM machine (3DLAM LLC, St. Petersburg, Russia) in an argon atmosphere on a steel build platform. Modifications were made to the 3D printer’s factory model to produce multi-material samples. Modifications were made to the powder feeding system, including the incorporation of an additional hopper for the second material and a dosing device. Furthermore, modifications were made to the second material distribution system of the multi-material module. The process parameters of the multi-material samples are presented in Table 1.

**Figure 2:**
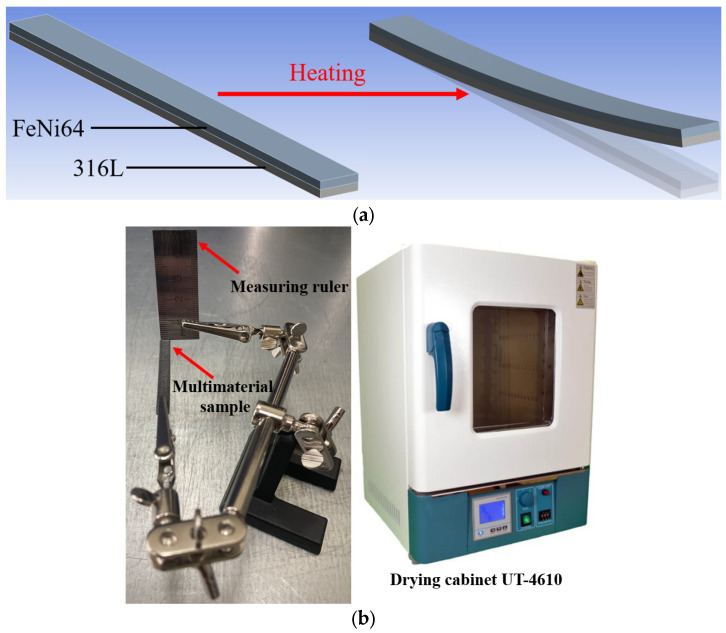
The 316L/FeNi36 multi-material sample and functional testing equipment: (**a**) realization of the shape-changing effect; (**b**) functional testing equipment.

The presence of two materials with different CTE within the same part enables the controlled shape-changing. The CTE of 316L is approximately 16 × 10^−6^ K^−1^, while FeNi36 exhibits a value of approximately 1.2 × 10^−6^ K^−1^ (at temperatures up to 100 °C). The difference in CTE between the two alloys results in a non-uniform change in linear dimensions when heated. By selecting the geometry and distribution of alloys in a multi-material product, it is possible to predictably change shape upon heating. For example, a 316L/FeNi36 multi-material can be used as an actuator, gripper, mechanical switcher, etc. For functional studies, the simplest variant of sample geometry was considered. The functional test samples were plates with a width of 5 mm and a length of 60 mm. The height of the plates was varied: (1) 250 µm FeNi36 and 500 µm 316L (sample 250/500); (2) 500 µm FeNi36 and 500 µm 316L (sample 500/500); (3) 500 µm FeNi36 and 1000 µm 316L (sample 500/1000). The representation of the shape-changing principle in multi-material samples under the influence of temperature is presented in Figure 2a.

A UT-4610 drying cabinet (ULAB, Nanjing, China) with a heating temperature of 300 °C, a sample holder, and a measuring ruler were used to perform functional tests on the 316L/FeNi36 multi-material samples (Figure 2b). A video camera was utilized to analyze the correlation between the heating temperature and the sample movement. The measurement accuracy of the shape-changing effect is not more than 500 μm.

### 2.3. Computational Model for the Functional Testing of the 316L/FeNi36 Multi-Material Samples

A computer simulation was conducted using the ANSYS 19 R2 software. The properties of the alloys were obtained from the software’s material library. It was assumed that the properties of the alloys were similar, except for the CTE, which was different for the two alloys. The CTE was found to vary with temperature. The finite element mesh consisted of hexahedrons, with the connection between the two alloys established through the ‘bonded’ type (the presence of an interfacial zone was not considered). The elastic zone of the samples was investigated. Uniform heating with equal steps up to 270 °C was carried out (without exposure). It should be noted that the model is based on several assumptions due to the fact that modeling is not the main objective of this work, and it is presented for the purpose of preliminary assessment of the potential feasibility of its use.

### 2.4. Post-Treatment and Characterization 

To achieve a more uniform composition and properties in the multi-material samples, a heat treatment was conducted in a vacuum furnace (Carbolite Gero GmbH & Co., KG, Neuhausen, Germany) according to the following parameters: heating up to 1050 °C; maintaining the temperature for two hours; and then cooling in the furnace. The treatment was carried out in an inert gas environment. The microstructure of the 316L/FeNi36 multi-material samples was examined using a Leica DMi8 M optical microscope (Leica Microsystems, Wetzlar, Germany). The samples were etched using aqua regia, a solution of nitric acid (HNO_3_) and hydrochloric acid (HCl) in a 3:1 ratio. The chemical composition was studied using a Mira 3 scanning electron microscope equipped with an energy-dispersive X-ray spectroscopy module (TESCAN, Brno, Czech Republic). The phase composition was analyzed on a Rigaku SmartLab (CuKa radiation, Rigaku Corporation, Tokyo, Japan). Microhardness was determined using a Vickers MicroMet 5101 microhardness tester (Buehler Ltd., Lake Bluff, IL, USA).

## 3. Results and Discussion

### 3.1. Porosity and Microstructure of the 316L/FeNi36 Multi-Material Samples

**Figure 3:**
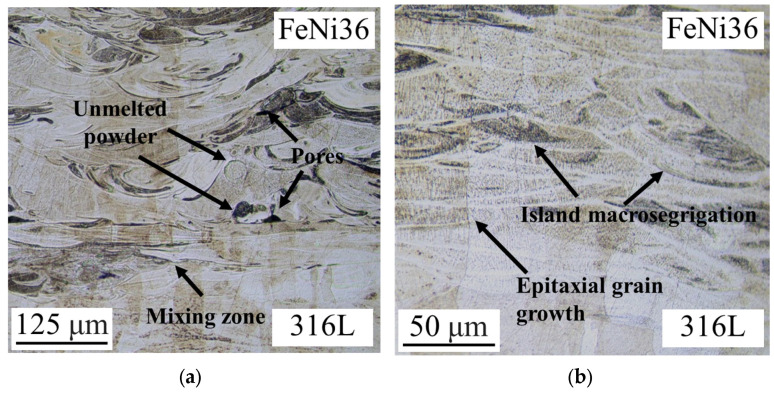
Results of defects and microstructure studies in the interfacial zone of the 316L/FeNi36 multi-material: (**a**) defects analysis; (**b**) microstructure analysis.

The porosity analysis of the 316L/FeNi36 multi-material samples reveals the absence of defects in the 316L zone (Figure 3a). In the interfacial zone, there are areas with the two alloys mixing, but no defects are observed (Figure 3a). The FeNi36 zone shows the presence of porosity, undissolved particles, and an island macrosegregation. A more detailed examination of the multi-material samples reveals that the microstructure consists of large epitaxial growth grains, which propagate in a directional manner from the 316L alloy through the interfacial zone to the FeNi36 region (Figure 3b). It is also noteworthy that the interfacial zone exhibits no distinguishing boundary between the two alloys, as they share a common primary component (Fe) and similar other components (Ni).

The island macrosegregation phenomenon observed in the zone of the FeNi36 composition can be explained by the Marangoni effect [27]. The Marangoni effect is a phenomenon that occurs when an elevated temperature in the central region of the melt pool causes a decrease in the surface tension, resulting in the molten metal flowing in the opposite direction. The continuous energy input serves to enhance the reverse flow, which then returns to the center of the melt pool, resulting in the formation of swirls [28]. As a result of the rapid cooling and the insufficient time for the distribution of chemical elements, inhomogeneities emerge that result in the formation of island macrosegregation in swirls [29]. A more detailed discussion of this feature can be found in the following section.

### 3.2. Chemical and Phase Composition of the 316L/FeNi36 Multi-Material Samples

**Figure 4:**
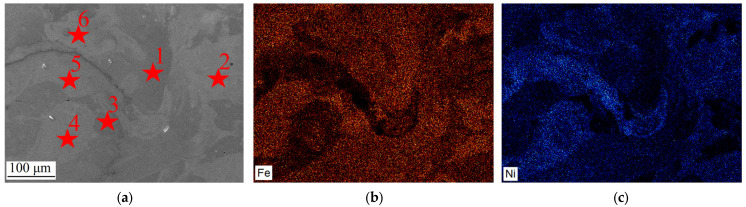
The results of the chemical composition study in the FeNi36 zone: (**a**) investigated area; (**b**) Fe distribution; (**c**) Ni distribution.

Figure 4 illustrates element maps in the FeNi36 composition zone. It can be observed that the chemical composition of this zone exhibits a notable degree of heterogeneity, which can be attributed to the fact that the FeNi36 composition was obtained by the mixing of two powders. It can be observed that there are regions with greater enrichment in Fe (Figure 4b) and Ni (Figure 4c). Additionally, there are regions with an average chemical composition of the two elements.

A more detailed analysis of the chemical element distribution in the FeNi36 zone reveals the presence of several distinct areas (Table 2). The chemical compositions in points 2 and 5–6 are in accordance with the composition of the FeNi36 alloy. In some areas, the concentration of iron is higher compared to other elements (point 1). In different areas, the ratio of the two elements is approximately equal (point 4). Furthermore, undissolved particles of Fe are present at point 3. The presence of regions with a composition different from the FeNi36 alloy is undesirable. Nevertheless, there are numerous areas of similar composition (i.e., lighter areas), which can mitigate the negative impact of an inhomogeneous elements’ distribution.

**Figure 5:**
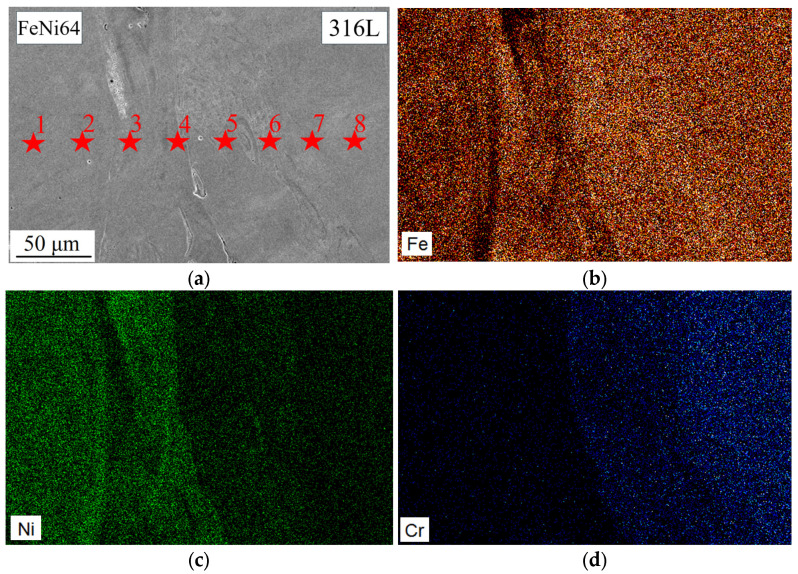
The results of the chemical composition study in the 316L/FeNi36 multi-material sample: (**a**) investigated area; (**b**) Fe distribution; (**c**) Ni distribution; (**d**) Cr distribution.

The results of the chemical composition studies in the 316L/FeNi36 multi-material sample are presented in Figure 5 and Table 3. It can be observed that the study area can be divided into three different zones. Zone 1 is the region comprising the FeNi36 composition (points 1–4), exhibiting a composition closely aligned with the FeNi36 alloy. Zone 2 is an interfacial zone (points 5–6) that exhibits an element inherent to 316L, specifically chromium, but in quantities below the expected levels for the alloy. Zone 3, which contains approximately 18% Cr and 14% Ni, corresponds to the chemical composition of the alloy and is, therefore, identified as the 316L zone (points 7–8). The interfacial zone was measured visually in accordance with the chemical composition analysis of the multi-material, and its size was found to be approximately 50 μm.

It is important to note that the presence of an interfacial zone in multi-materials has a dual effect. On the one hand, the formation of this zone reduces the stress concentration between two alloys, which is inherent in coatings or composites with a sharp interface between materials. On the other hand, the formation of this zone can lead to the formation of new phases, which is not always desirable. Furthermore, the formation of an undefined chemical composition zone can influence the mechanical properties of multi-material samples. The presence of an interfacial zone in the investigated multi-material sample will have an impact on the functional properties. The FeNi36 alloy is highly sensitive to chemical composition due to low CTE. Even an insignificant alteration in Ni composition can result in a considerable change in CTE.

The phase composition analysis of the 316L/FeNi36 multi-material sample indicates the presence of a phase inherent to 316L–γ-Fe. Furthermore, a sufficient quantity of the γ-Fe64Ni36 phase is observed, the presence of which was previously discussed in relation to the chemical composition analysis. The identification of this phase is a positive outcome, as its presence suggests that the desired phase composition was achieved during the SLM process. It is important to note that the multi-material sample also contains α-Fe. This phenomenon can be attributed to the non-uniform distribution of chemical composition within the FeNi36 region, which results in the formation of a zone with an incrementally higher Fe content.

**Figure 6:**
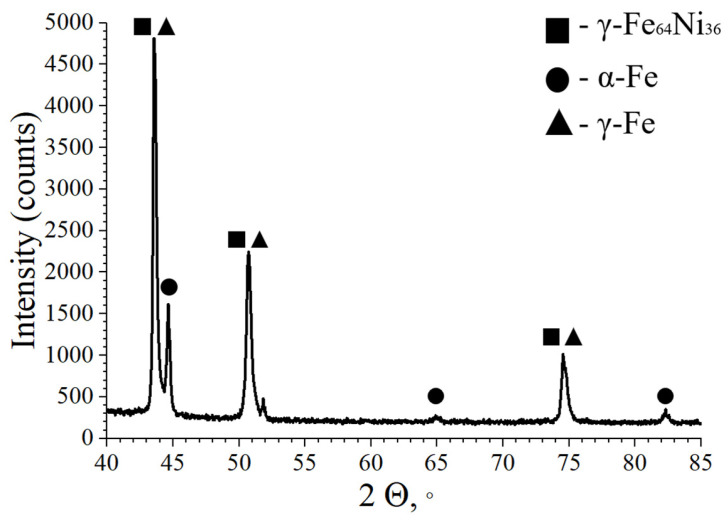
The results of the phase composition study in the 316L/FeNi36 multi-material sample (The powder diffraction files for FeNi36, α-Fe and γ-Fe see in Supplementary File S1).

In the context of phase formation in the Fe-Ni-Cr ternary system, it is important to note the existence of an intermediate phase Ni_2_Cr, which is formed in the solid state within the Cr–Ni system. The formation of the intermetallic compound FeNi_3_ with a Ni content of 74 at.% in the Fe-Ni system was confirmed. In the double system, a broad region of γ-solid solutions is identified (including γ-Fe64Ni36). In the Fe–Cr system, the formation of solid solutions with a bcc lattice is observed. The aforementioned phases are not present in the multi-material sample in accordance with phase composition analysis (Figure 6). This can be attributed to a positive component, as the presence of these phases could result in the formation of defects.

### 3.3. Hardness of the 316L/FeNi36 Multi-Material Samples

The results of the microhardness tests in the 316L/FeNi36 multi-material sample are presented in Table 4. The hardness in the FeNi36 zone (points 1–4) is approximately 163 HV, which is a similar value to the alloy (approximately 150 HV [30]). Subsequently, an interfacial zone with a hardness value of approximately 200 HV (points 5–6) is observed. This value is more closely aligned with the hardness of the 316L zone—approximately 214 HV (points 7–8). This hardness value is in close alignment with the data for 316L presented in the literature (222 HV [31]). The absence of a notable increase in hardness in the interfacial zone may indicate that no new phases or compounds with hardness values exceeding 316L are formed in this region. Furthermore, the similarity between the hardness observed in the interfacial and 316L zones may be evidence of a defect-free manufacturing process.

### 3.4. Functional Properties and Computer Simulation of the 316L/FeNi36 Multi-Material Samples

**Figure 7:**
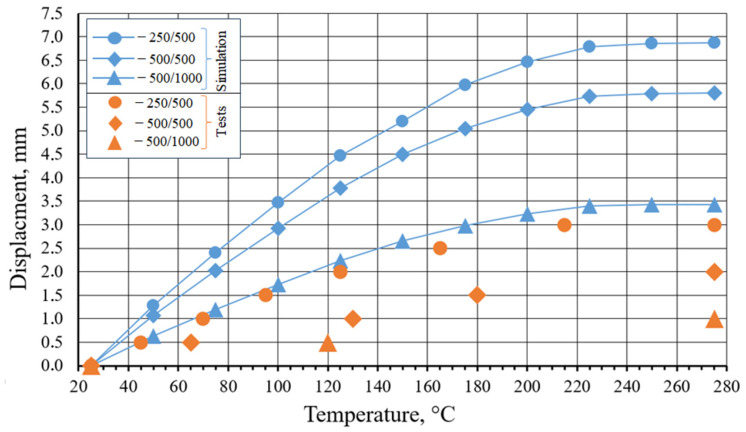
The results of the functional properties tests and computer simulation for the 316L/FeNi36 multi-material samples.

Figure 7 illustrates the results of functional tests conducted on the 316L/FeNi36 multi-material samples. The maximum displacement observed for the 250/500 sample was 3 mm; for the 500/500 sample, it was 2 mm, and for the 500/1000 sample, it was 1 mm. It is, therefore, reasonable to assume that an increase in the overall thickness of the sample has a negative impact on its functional properties. This may be attributed to the increase in sample stiffness, which appears to prevent the ability to achieve the shape-changing effect.

A more detailed analysis of the correlation between displacement and temperature reveals several dependencies. The temperature range between 25 °C and 125 °C exhibits the most pronounced shape-changing effect. The reason is that in this temperature range, the CTE of FeNi36 differs most significantly from the CTE of 316L. Following the temperature range of 125 °C to 215 °C, a reduction in the shape-changing effect is observed, which relates to a decrease in the discrepancy between the CTEs of the two alloys. Upon reaching 215 °C, the sample exhibits minimal change in dimension. The selected testing method and low displacement of the 500/500 and 500/1000 specimens do not permit precise behavior descriptions, so comparisons between samples are hard. Nevertheless, based on the behavior of the 250/500 sample, it can be seen that the shape-changing effect in multi-material samples is directly dependent on the CTE of FeNi36. For achieving the most pronounced shape-changing effect, the temperature range of 25–215 °C is more suitable.

Computer simulation can be used to facilitate the design of multi-material structures. In this paper, we present the preliminary findings of a computer simulation process in the ANSYS software. It should be noted that at this stage of the research, only the engineering approach in computer simulation was employed, and the initial assessment of the computer simulation suitability was conducted.

Figure 7 illustrates the results of the computer simulation in the heating process of the 316L/FeNi36 multi-material sample. It is evident that there is a notable divergence between the simulation outcomes and the actual experimental results: 56%, 66%, and 71% for samples 250/500, 500/500, and 500/1000 (for final displacement). The discrepancy can be explained by the influence of two factors. First, the zone of FeNi36 was obtained by mixing elemental powders, and, as demonstrated in previous studies, it exhibits inhomogeneities in chemical composition. This aspect is not considered in the computational model and assumes that the material has a homogeneous chemical composition and properties. Second, the multi-material exhibits an interfacial zone (approximately 50 µm) with an unspecified chemical composition. This aspect is also not taken into account by the computational model, which consequently affects the discrepancy between the simulation outcomes and the actual experimental results. The authors make the suggestion that, in order to potentially reduce the divergence between the modeling and the actual experiment, it may be an effective approach to specify the properties of the FeNi36 zone with greater precision and to consider the features of the interfacial zone. Nevertheless, further research is required to confirm this assumption. The increase in discrepancy for samples 250/500 and 500/1000 can be attributed to the quantity of material utilized in the simulation process, which increased.

## 4. Conclusions

It is possible to create smart bio-inspired materials through 4D printing using the SLM. The objective of this study was to examine the 316L/FeNi36 multi-material with a bio-inspired structure. The microstructure, chemical and phase compositions, and hardness of the multi-material samples were investigated. Moreover, functional tests and computer simulations of the shape-changing effect were also carried out. The following results were obtained:

1. In the interfacial zone of the 316L/FeNi36 multi-material, areas between the two alloys are observed without any evidence of defects. The microstructure consists of large grains with epitaxial growth, which propagate in a directional manner from the 316L alloy through the interfacial zone to the FeNi36 region. The FeNi36 zone shows the presence of porosity, undissolved particles, and an island macrosegregation;
2. The 316L/FeNi36 multi-material sample has three distinct zones of chemical composition: the FeNi36 zone; the interfacial zone; and the 316L zone. The size of the interfacial zone is approximately 50 µm. The zone of FeNi36 exhibits a pronounced heterogeneity in chemical composition. The phase composition analysis of the 316L/FeNi36 multi-material sample indicates the presence of a phase inherent to 316L–γ-Fe. The hardness in the FeNi36 zone is approximately 163 HV, followed by an interfacial zone with a hardness of approximately 200 HV and then the 316L zone with a hardness of approximately 214 HV;
3. The maximum displacement observed for the 250/500 sample was 3 mm; for the 500/500 sample, it was 2 mm, and for the 500/1000 sample, it was 1 mm. The shape-changing effect in multi-material samples is directly dependent on the CTE of FeNi36. For achieving the most pronounced shape-changing effect, the temperature range of 25–215 °C is more suitable. There is a notable divergence between the simulation outcomes and the actual experimental results. The discrepancy can be explained by the presence of assumptions in the computational model (the presence of a homogeneous chemical composition in the FeNi36 zone and the absence of an interfacial zone).

## Figures and Tables

**Table 1:** L-PBF process parameters for fabrication of 316L/FeNi36 multi-material samples.

Alloy	Scanning Speed, mm/s	Laser Power, W	Hatch Distance, μm	Later Thickness, μm	Volume Energy Density, J/mm^3^
316L	760	275	100	50	72.37
FeNi36	250	800	60	30	174.61

**Table 2:** Chemical composition in the FeNi36 zone.

Numbers from Figure 4	Amount of Fe, %	Amount of Ni, %
1	78.05	21.95
2	36.74	63.26
3	96.37	3.63
4	55.87	44.13
5	35.98	64.02
6	35.10	64.90

**Table 3:** Chemical composition in the 316L/FeNi36 multi-material sample.

Numbers from Figure 5	Amount of Fe, %	Amount of Ni, %	Amount of Cr, %
1	63.15	36.85	
2	47.13	52.87	
3	62.58	37.42	
4	65.10	34.90	
5	68.66	24.51	5.54
6	68.39	22.41	7.99
7	64.42	14.31	17.97
8	65.13	14.24	17.92

**Table 4:** The results of the microhardness analysis in the 316L/FeNi36 multi-material sample.

Numbers from Figure 5	Microhardness, HV
1	159
2	164
3	163
4	168
5	199
6	201
7	215
8	212

## Data Availability

The main data are provided in this paper. Any other raw/processed data required to reproduce the findings of this study are available from the corresponding author upon request.

## Supplementary Materials

The following supporting information can be downloaded at https://www.mdpi.com/article/10.3390/mi15111288/s1, File S1: The powder diffraction files for FeNi36, α-Fe and γ-Fe.
