# Supplementary material for: Investigation of the 4D Multi-Material 316L/FeNi36 Obtained by Selective Laser Melting
Source: Micromachines (Basel). 2024 Oct 23;15(11):1288. doi: 10.3390/mi15111288 (PMC11596448; doi:10.3390/mi15111288)
Supplement: Supplementary file 1 [file micromachines-15-01288-s001.zip › micromachines-3252666-supplementary.pdf]

**Status** Primary **Quality Mark:** Indexed **Environment:** Ambient **Temp:** 298.0 K (Assigned by ICDD editor)
**Chemical Formula:** Fe0.64 Ni0.36 **Empirical Formula:** Fe0.64 Ni0.36 **Weight %:** Fe62.85 Ni37.15
**Atomic %:** Fe64.00 Ni36.00 **Compound Name:** Iron Nickel **Entry Date:** 09/01/1997

**Radiation:** CoKα (1.7902 Å) **Filter:** Graph Mono **d-Spacing:** Diffractometer **Intensity:** Diffractometer - Peak

**Crystal System:** Cubic **SPGR:** Fm-3m (225)
**Author's Unit Cell [ a:** 3.5922(1) Å **Volume:** 46.35 Å³ **Z:** 4.00 **MolVol:** 11.59 **]** **Calculated Density:** 8.149 g/cm³
**SS/FOM:** F(5) = 42.8(0.0234, 5)

**Space Group:** Fm-3m (225) **Molecular Wt:** 56.87 g/mol
**Crystal Data [ a:** 3.592 Å **b:** 3.592 Å **c:** 3.592 Å **α:** 90.00° **β:** 90.00° **γ:** 90.00° **XtlCell Vol:** 46.35 Å³
**XtlCell Z:** 4.00 **a/b:** 1.000 **c/b:** 1.000 **]**
**Reduced Cell [ a:** 2.540 Å **b:** 2.540 Å **c:** 2.540 Å **α:** 60.00° **β:** 60.00° **γ:** 60.00° **RedCell Vol:** 11.59 Å³ **]**

**Crystal (Symmetry Allowed):** Centrosymmetric

**Subfiles:** Inorganic, Metal & Alloy **Pearson Symbol:** cF4.00 **Prototype Structure (Formula Order):** Cu
**Prototype Structure (Alpha Order):** Cu **LPF Prototype Structure (Formula Order):** Cu,cF4,225
**LPF Prototype Structure (Alpha Order):** Cu,cF4,225 **ANX:** N

**Cross-Ref PDF #'s:** 04-002-1863 (Experimental <-> LPF), 04-002-1864 (Experimental <-> LPF), 04-002-8941 (Experimental <-> LPF), 04-003-5440 (Experimental <-> LPF), 04-006-6404 (Experimental <-> LPF), 04-006-6665 (Experimental <-> LPF)

**References:**

| Type | DOI | Reference |
|---|---|---|
| Primary Reference | | Samvel`yan, R., Abovyan, E., Agbalyan, S., Manukyan, N., Sakanyan, M. Soviet Powder Metall. and Met. Ceramics 1991, 30, 606. |

**Database Comments:** ANX: N. Sample Preparation: Formed by mechanical alloying of bimetallic layers. Warning: Not enough reflections above the intensity cut off to meet higher quality mark criteria. Unit Cell Data Source: Powder Diffraction.

**d-spacings (5) - Fe0.64 Ni0.36 - 00-047-1405 (Stick, Fixed Slit Intensity) - X-ray (Cu Kα1 1.54056 Å)**

| 2θ (°) | d (Å) | I | h | k | l | * |
|---|---|---|---|---|---|---|
| 43.604 | **2.0740** | 100 | 1 | 1 | 1 | |
| 50.794 | **1.7960** | 49 | 2 | 0 | 0 | |
| 74.677 | **1.2700** | 25 | 2 | 2 | 0 | |
| 90.630 | 1.0834 | 20 | 3 | 1 | 1 | |
| 95.904 | 1.0373 | 7 | 2 | 2 | 2 | |

**© 2024 International Centre for Diffraction Data. All rights reserved.**

**Status** Alternate **Quality Mark:** Indexed **Environment:** Non-ambient Temperature **Temp:** 318.0 K **Phase:** α
**Chemical Formula:** Fe **Empirical Formula:** Fe **Weight %:** Fe100.00 **Atomic %:** Fe100.00
**Compound Name:** Iron **Alternate Name:** α-Fe **Entry Date:** 09/01/2013 **Modification Date:** 09/01/2020
**Modifications:** Update

**Radiation:** CuKα1 (1.5406 Å) **d-Spacing:** Calculated **Intensity:** Calculated - Peak

**Crystal System:** Cubic **SPGR:** Im-3m (229)
**Author's Unit Cell [ a:** 2.869(1) Å **Volume:** 23.62 $Å^3$ **Z:** 2.00 **MolVol:** 11.81 **]** **Calculated Density:** 7.854 $g/cm^3$
**Structural Density:** 7.852 $g/cm^3$ **SS/FOM:** F(6) = 999.9(0.0002, 6) **I/Ic:** 10.77

**Space Group:** Im-3m (229) **Molecular Wt:** 55.85 g/mol
**Crystal Data [ a:** 2.869 Å **b:** 2.869 Å **c:** 2.869 Å **α:** 90.00° **β:** 90.00° **γ:** 90.00° **XtlCell Vol:** 23.62 $Å^3$
**XtlCell Z:** 2.00 **a/b:** 1.000 **c/b:** 1.000 **]**
**Reduced Cell [ a:** 2.485 Å **b:** 2.485 Å **c:** 2.485 Å **α:** 109.47° **β:** 109.47° **γ:** 109.47° **RedCell Vol:** 11.81 $Å^3$
**]**

**Crystal (Symmetry Allowed):** Centrosymmetric

**Subfiles:** Common Phase, Forensic, Hydrogen Storage Material, Inorganic, Metal & Alloy **Pearson Symbol:** cI2.00
**Prototype Structure (Formula Order):** W **ANX:** N **Wyckoff Sequence:** a (IM-3M)

**References:**

| Type | DOI | Reference |
|---|---|---|
| Primary Reference | | Calculated from ICSD using POWD-12++. |
| Structure | 10.1016/j.jallcom.2011.03.147 | Crisan, O., Crisan, A.D. "Phase transformation and exchange bias effects in mechanically alloyed Fe/magnetite powders". J. Alloys Compd. 2011, 509 (23), 6522. |

**Database Comments:** ANX: N. Analysis: Fe1. Formula from original source: Fe. ICSD Collection Code: 180969. Calculated Pattern Original Remarks: Structure determination using synchrotron radiation (powder). Structure refinement by Rietveld method. At 373 K: a=2.870(1). At 473 K: a=2.875(5). At 573 K: a=2.882(4). Phase appearing in mechanically alloyed Fe/magnetite powders. Temperature of Data Collection: 318 K. Minor Warning: No R factors reported/abstracted. Wyckoff Sequence: a (IM-3M). Unit Cell Data Source: Powder Diffraction.

**d-spacings (6) - Fe - 01-080-3816 (Stick, Fixed Slit Intensity) - X-ray (Cu Kα1 1.54056 Å)**

| 2θ (°) | d (Å) | I | h | k | l | * | 2θ (°) | d (Å) | I | h | k | l | * |
|---|---|---|---|---|---|---|---|---|---|---|---|---|---|
| 44.629 | **2.028690** | 1000 | 1 | 1 | 0 | | 98.821 | 1.014340 | 46 | 2 | 2 | 0 | |
| 64.955 | **1.434500** | 116 | 2 | 0 | 0 | | 116.211 | 0.907257 | 63 | 3 | 1 | 0 | |
| 82.242 | **1.171260** | 177 | 2 | 1 | 1 | | 136.887 | 0.828209 | 17 | 2 | 2 | 2 | |

**© 2024 International Centre for Diffraction Data. All rights reserved.**

**Status** Primary **Quality Mark:** Star **Environment:** Ambient **Temp:** 298.0 K (Assigned by ICDD editor)
**Chemical Formula:** Fe0.49 Cr0.29 Ni0.16 C0.06 **Empirical Formula:** C0.06 Cr0.29 Fe0.49 Ni0.16
**Weight %:** C1.37 Cr28.69 Fe52.07 Ni17.87 **Atomic %:** C6.00 Cr29.00 Fe49.00 Ni16.00
**Compound Name:** Chromium Iron Nickel Carbon **Alternate Name:** 304-stainless steel, austenite
**Entry Date:** 09/01/1983 **Modification Date:** 09/01/2018 **Modifications:** Formula

**Radiation:** CuKα (1.5419 Å) **Internal Standard:** Si **d-Spacing:** Diffractometer **Intensity:** Diffractometer - Peak

**Crystal System:** Cubic **SPGR:** Fm-3m (225)
**Author's Unit Cell [ a:** 3.5911(1) Å **Volume:** 46.31 Å³ **Z:** 4.00 **MolVol:** 11.58 **]** **Calculated Density:** 7.538 g/cm³
**Color:** Black **SS/FOM:** F(6) = 56.6(0.0177, 6)

**Space Group:** Fm-3m (225) **Molecular Wt:** 52.55 g/mol
**Crystal Data [ a:** 3.591 Å **b:** 3.591 Å **c:** 3.591 Å **α:** 90.00° **β:** 90.00° **γ:** 90.00° **XtlCell Vol:** 46.31 Å³
**XtlCell Z:** 4.00 **a/b:** 1.000 **c/b:** 1.000 **]**
**Reduced Cell [ a:** 2.539 Å **b:** 2.539 Å **c:** 2.539 Å **α:** 60.00° **β:** 60.00° **γ:** 60.00° **RedCell Vol:** 11.58 Å³ **]**

**Crystal (Symmetry Allowed):** Centrosymmetric

**Subfiles:** Inorganic, Metal & Alloy **Pearson Symbol:** cF4.00 **Prototype Structure (Formula Order):** Cu
**Prototype Structure (Alpha Order):** Cu **LPF Prototype Structure (Formula Order):** Cu,cF4,225
**LPF Prototype Structure (Alpha Order):** Cu,cF4,225

**References:**

| Type | DOI | Reference |
|---|---|---|
| Primary Reference | | Pfoertsch, Ruud, Penn State University, University Park, Pennsylvania, USA. ICDD Grant-in-Aid 1982. |

**Database Comments:** Analysis: Quantitative analysis by Atomic Absorption Spectroscopy: chromium 17.9%, nickel 11.4%, molybdenum <0.01%, silicon 0.88%, analysis incomplete. General Comments: Austenitic steel. Synthetic taenite is "Ni"-"Fe" rich analog.

**d-spacings (6) - Fe0.49 Cr0.29 Ni0.16 C0.06 - 00-033-0397 (Stick, Fixed Slit Intensity) - X-ray (Cu Kα1 1.54056 Å)**

| 2θ (°) | d (Å) | I | h | k | l | * | 2θ (°) | d (Å) | I | h | k | l | * | 2θ (°) | d (Å) | I | h | k | l | * |
|---|---|---|---|---|---|---|---|---|---|---|---|---|---|---|---|---|---|---|---|---|
| 43.582 | **2.0750** | 100 | 1 | 1 | 1 | | 74.697 | 1.2697 | 26 | 2 | 2 | 0 | | 95.965 | 1.0368 | 12 | 2 | 2 | 2 | |
| 50.791 | **1.7961** | 45 | 2 | 0 | 0 | | 90.694 | **1.0828** | 30 | 3 | 1 | 1 | | 118.156 | 0.8979 | 3 | 4 | 0 | 0 | |

© 2024 International Centre for Diffraction Data. All rights reserved.
